# Microbial characterization and fermentative characteristics of crop maize ensiled with unsalable vegetables

**DOI:** 10.1038/s41598-019-49608-w

**Published:** 2019-09-12

**Authors:** Kristian Hooker, Daniel L. Forwood, Eleonora Caro, Yuxin Huo, Devin B. Holman, Alex V. Chaves, Sarah J. Meale

**Affiliations:** 1 School of Life and Environmental Sciences, Faculty of Science, University of Sydney, Camperdown, NSW Australia; 2 Department of Agricultural, Forestry and Food Science, University of Turin, Torino, TO Italy; 3 Lacombe Research and Development Centre, Agriculture and Agri-Food Canada, Lacombe, AB Canada; 4 School of Agriculture and Food Sciences, Faculty of Science, University of Queensland, Gatton, QLD Australia

**Keywords:** High-throughput screening, Applied microbiology

## Abstract

Incorporation of carrot or pumpkin at 0, 20 or 40% dry matter (DM-basis) with crop maize, with or without a silage inoculant was evaluated after 70 days ensiling for microbial community diversity, nutrient composition, and aerobic stability. Inclusion of carrots or pumpkin had a strong effect on the silage bacterial community structure but not the fungal community. Bacterial microbial richness was also reduced (P = 0.01) by increasing vegetable proportion. Inverse Simpson’s diversity increased (P = 0.04) by 18.3% with carrot maize silage as opposed to pumpkin maize silage at 20 or 40% DM. After 70 d ensiling, silage bacterial microbiota was dominated by *Lactobacillus* spp. and the fungal microbiota by *Candida tropicalis, Kazachstania humilis* and *Fusarium denticulatum*. After 14 d aerobic exposure, fungal diversity was not influenced (P ≥ 0.13) by vegetable type or proportion of inclusion in the silage. Inoculation of vegetable silage lowered silage surface temperatures on day-7 (P = 0.03) and day-14 (P ≤ 0.01) of aerobic stability analysis. Our findings suggest that ensiling unsalable vegetables with crop maize can successfully replace forage at 20 or 40% DM to produce a high-quality livestock feed.

## Introduction

Despite heightening concerns over food security, up to 1.6 Gt of food is wasted globally each year^1,2^. Within Australia, ~40% of vegetables are rejected by commercial grading standards due to cosmetic defects, regardless of their nutritional quality or suitability for consumption. This amounts to 1.3 billion tonnes of food waste and is estimated to cost Australia $8.4 billion annually^3,4^. Current post-grading avenues for unsalable vegetables include incineration, composting and biofuel production. However, in developing countries, food waste often enters landfills and waterways^5^. Consequently, considerable environmental damage is caused through their rapid decomposition and resultant greenhouse gas production^6^. Thus, necessitating an alternative avenue for unsalable vegetables.

Previous studies have identified unsalable vegetables as potential feed additives in livestock diets due to their high palatability, high concentration of total digestible nutrients^7^, and antioxidant properties^8^. Recently, the replacement of common feed additives, wheat bran or soybean meal, with vegetables sourced from markets at up to 275 g kg^−1^ DM in growing bull diets resulted in similar rumen degradability^9^. Additionally, as the crude protein values range between 5% and 18% dry matter (DM), and with energy values ranging from 8.8 MJ/Kg DM in pumpkins, up to 13.8 MJ/Kg DM in carrots, it is expected that they can be effectively integrated in ruminant diets to support targeted growth rates. Yet, due to their high moisture content of 30%, a preservation method is required to extend their shelf life.

Identification of the ideal ensiling ratio and crop selection is paramount. Here, we investigated the use of maize. Previously, ensiled crop maize successfully replaced grass silage and was associated with increased feed intake, utilisation of metabolizable energy and improvements in carcase weight gain of crossbred beef cattle^10^. Prior to ensiling vegetables, consideration must be given to the effect of sanitising agents commonly used in vegetable processing plants on microbial community profiles. For example, sodium hypochlorite (NaClO) is damaging to cellular transport mechanisms in pathogens such as *Escherichia coli*^11^ and has been shown to reduce the proliferation and abundance of pathogenic bacteria, yeasts, moulds and spoilage microbes in carrot peels^12^. To overcome this, the use of silage inoculant will be investigated. Previous literature suggests the use of vegetable residues, with or without a lactic acid-bacteria (LAB) inoculant, in a crop silage can produce a highly digestible feed that positively affects the profile of acetic and propionic acids^5,13^.

Therefore, the objectives of this study were to determine whether unsalable vegetables at varying proportions could be successfully ensiled with crop maize to produce a silage of high nutritional value, which maintains aerobic stability, and quality through *in vitro* fermentation, and to characterise the bacterial and fungal microbiota of the resulting silage. We hypothesised that fresh, unsalable vegetables ensiled with crop maize would produce a silage with greater acetic and propionic acid concentrations, increase the overall microbial diversity at higher vegetable levels, and improve aerobic stability with the introduction of a second-generation microbial inoculant in the vegetable silage.

## Results

### Chemical composition of silage

The chemical composition of maize at harvest, in addition to carrot and pumpkin are presented in Supplementary Table 1. The dry matter (DM) content of maize silage was affected (P = 0.02) by the interaction of vegetable type × level (Table 1). The DM content of 100% ensiled maize was greater (P = 0.02) than with the addition of 40% of either vegetable, or 20% pumpkin, but was similar to 20% carrot inclusion. When comparing across vegetable type, the inclusion of pumpkin resulted in wetter (P = 0.02) silage, compared to carrots at both 20 and 40% DM inclusion rates. Similarly, DM loss (g) increased linearly (P = 0.01) with increasing amounts of vegetable in the silage.

**Table 1:** Physio-chemical characteristics, nutrient composition, and organic acid profile of maize ensiled with unsalable vegetables after 70 days of ensiling.

Parameter^1^	0%	Carrots		Pumpkin		SEM	Veg	P-values^2^			
		20%	40%	20%	40%			Level	Veg × Level	Linear	Quadratic
Dry matter content, %	37.00a	35.30a	31.00b	29.00b	22.40c	1.49	<0.01	<0.01	0.02	<0.01	0.79
Dry matter loss, g	62.50	77.50	78.80	82.50	98.80	7.84	0.24	0.02	0.47	0.01	0.53
Silage pH	3.80	3.90	4.00	3.90	3.90	0.09	0.70	0.18	0.84	0.10	0.53
Ash, % DM	4.90b	5.40a	5.40a	5.20b	5.90a	0.14	0.33	<0.01	0.05	<0.01	0.74
NDF, % in DM	44.60	41.60	44.10	43.50	43.30	1.43	0.76	0.39	0.62	0.55	0.22
CP, % in DM	6.30	7.80	8.10	7.80	8.50	n/a	n/a	n/a	n/a	n/a	n/a
Crude fat, % in DM	3.00	3.60	3.50	3.50	3.90	0.19	0.57	0.01	0.4	<0.01	0.29
NFC, % in DM	41.20	41.60	38.90	40.00	38.40	n/a	n/a	n/a	n/a	n/a	n/a
***Volatile fatty acids, mM***											
Acetic acid	13.60	19.80	18.40	22.00	22.50	0.88	0.01	<0.01	0.10	<0.01	<0.01
Propionic acid	0.32b	0.25b	0.38b	0.66a	0.31b	0.08	0.13	0.25	0.03	0.73	0.11
Butyric acid	0.09	0.09	0.10	0.10	0.12	0.01	0.13	0.09	0.40	0.04	0.35
Total VFA	14.70	20.30	19.10	23.00	23.10	0.87	0.01	<0.01	0.11	<0.01	<0.01
***Organic acid concentration, mM***											
Succinic acid	0.27	0.57	0.66	0.49	0.89	0.18	0.74	0.12	0.73	0.05	0.95
Lactic acid	8.60	4.76	9.60	5.08	2.88	1.63	0.17	0.10	0.18	0.21	0.14
Ethanol, %	0.11	0.11	0.12	0.13	0.13	0.0070	0.18	0.26	0.49	0.14	0.50

^1^NDF = Neutral detergent fiber; CP = Crude protein; NFC = non-fibrous carbohydrates, 100 − (CP + NDF + CF + ash).

^2^P-values: Vegetable, carrot or pumpkin; Level, proportion of vegetable in silage on DM basis; Vegetable × Level, interaction. Statistical significance declared at P ≤ 0.05.

Means with different letters – a, b, c differ (P ≤ 0.05).

The percentage of ash was affected by the interaction of vegetable × level (P = 0.05), where 20% and 40% carrot had similar concentrations of ash (5.4%) in the DM and were greater than the control (4.9%; Table 1). Conversely, silage containing pumpkin showed a linear (P ≤ 0.01) increase in the percentage of ash in the silage (Table 1). Concentrations of NDF (% in the DM) or silage pH were not affected (P ≥ 0.22) by the addition of either vegetable (Table 1). Crude fat concentration (% in the DM) increased linearly (P < 0.01) with increasing vegetable inclusion, regardless of the vegetable type. Statistical analysis of crude protein was not possible since only 1 sample per treatment was analyzed.

### Organic acid concentrations

Total volatile fatty acids (VFA) and concentrations of acetic acid (mM) were greater (P < 0.01) in silages containing pumpkin, compared to control or carrot treatments (Table 1). Additionally, a quadratic increase (P < 0.01) in both total VFA and acetic acid concentration were observed with increasing inclusion of carrot or pumpkin. The concentration of propionic acid (mM) in maize silage was affected by the interaction of vegetable × level (P = 0.03), where pumpkin inclusion at 20% DM increased propionic acid concentrations by 48%, compared to the control. Butyrate tended (P = 0.09) to increase with increasing inclusion of vegetables in the silage. However, no other effects were observed on organic acid concentrations.

### Silage aerobic stability

Surface temperature (°C) of maize was not affected by vegetable inclusion or vegetable type, except for a decline in surface temperature (P = 0.05) with increasing inclusion of vegetable on the second day of the 14-day aerobic stability trial (Table 2; Supplementary Table 2).

**Table 2:** Aerobic stability of maize silage after 70 days of ensiling, based on surface temperature (°C) of silage.

Day	0%	Carrots		Pumpkin		SEM	P-values^a^				
		20%	40%	20%	40%		Veg	Level	Veg × Level	Linear	Quadratic
1	22.96	22.96	22.8	22.84	22.76	0.13	0.63	0.38	0.91	0.17	0.8
2	22.76	22.53	22.54	22.28	22.29	0.16	0.2	0.05	0.67	0.04	0.2
3	23.88	23.93	23.35	23.75	23.81	0.16	0.51	0.18	0.18	0.09	0.43
4	22.81	22.83	22.44	22.5	22.66	0.22	0.86	0.49	0.46	0.24	0.93
5	23.82	23.84	23.41	23.77	23.74	0.25	0.67	0.55	0.71	0.32	0.63
6	23.72	23.71	23.73	23.59	23.6	0.39	0.79	0.98	0.98	0.89	0.89
7	23.6	23.65	23.55	23.71	23.82	0.21	0.53	0.91	0.79	0.71	0.84
8	22.71	22.74	22.59	22.42	22.5	0.3	0.58	0.84	0.87	0.58	0.85
9	23.49	23.45	23.39	23.19	23.36	0.35	0.73	0.89	0.93	0.74	0.71
10	23.45	23.3	23.24	23.27	23.41	0.22	0.8	0.73	0.88	0.56	0.59
11	25.03	25.03	24.94	24.84	24.92	0.29	0.76	0.92	0.94	0.72	0.84
12	24.84	24.84	24.72	24.76	24.62	0.55	0.9	0.95	1.00	0.75	0.92
13	23.91	24.01	23.89	23.86	24.02	0.54	0.99	1.00	0.97	0.93	1.00
14	23.47	23.31	23.42	23.18	23.43	0.14	0.72	0.27	0.86	0.73	0.12

^a^P-values for the treatments: Vegetable, carrot or pumpkin; Level, proportion of vegetable in silage on DM basis; Vegetable × Level, interaction. Statistical significance declared at P ≤ 0.05.

### Bacterial and fungal diversity and composition of silage

Alpha diversity measures for the original crop, silage at day 0 and day 14 after opening are presented in Table 3. A linear decrease (P = 0.01) in the number of bacterial operational taxonomic units (OTUs) was observed with increasing amounts of vegetable prior to ensiling. The addition of pumpkin and inoculant to the silage produced greater (P ≤ 0.003; 5.47 and 9.64%, respectively) diversity (as indicated by Shannon diversity), compared to silage with carrots and without inoculant. There was no effect of vegetable, or level on the inverse Simpson’s diversity index of bacteria in the maize crop prior to ensiling.

**Table 3:** Alpha diversity of the silage bacterial and fungal microbiota by treatments with increasing proportions of carrot or pumpkin.

	0	Carrots		Pumpkin		SEM	P-values^a^				
		20%	40%	20%	40%		Veg	Level	Veg × Level	L	Q
**Bacteria - Initial**											
Number of OTUs	33.8	28.3	26.5	29.8	29	1.59	0.33	0.01	0.74	<0.01	0.23
Shannon diversity	2.07	1.98	1.98	2.16	2.13	0.036	<0.01	0.85	0.06	0.69	0.69
Simpsons diversity	5.52	5.26	5.06	6.08	6.07	0.292	0.24	0.88	0.47	0.9	0.71
**Bacteria - Silage**											
Number of OTUs	55	61	55.8	52.3	37.3	4.41	0.03	0.09	0.15	0.08	0.15
Shannon diversity	2.55	2.79	2.44	2.58	2.22	0.347	0.66	0.69	0.94	0.59	0.5
Simpsons diversity	10.2	10	6.9	8.3	4.4	0.74	0.04	<0.01	0.26	<0.01	0.09
**Fungi - Silage**											
Number of OTUs	33	39	34.8	33.8	44.5	4.02	0.66	0.29	0.21	0.13	0.99
Shannon diversity	2.2	2.23	2.21	2.28	2.35	0.068	0.27	0.49	0.6	0.25	0.79
Simpsons diversity	5.25	5.62	5.65	5.9	5.67	0.426	0.78	0.47	0.94	0.36	0.43
**Fungi – Aerobic Stability**											
Number of OTUs	27.7	40.6	42.1	61.1	36.3	18.63	0.78	0.43	0.81	0.6	0.38
Shannon diversity	2.06	2.71	2.67	3.24	2.52	0.593	0.81	0.31	0.85	0.43	0.3
Simpsons diversity	5.8	10.1	9.1	14	12.1	3.72	0.53	0.37	0.85	0.33	0.37

^a^P-values for the treatments: Vegetable, carrot or pumpkin; Level, proportion of vegetable in silage on DM basis; Vegetable × Level, interaction. Statistical significance declared at P ≤ 0.05.

The number of bacterial and fungal OTUs and Shannon diversity were not affected (P ≥ 0.15) by treatments upon silage opening (Table 3). However, carrots incorporated into the maize silage had 18.3% greater (P = 0.04) inverse Simpsons diversity, compared to maize silage with pumpkin. A linear decrease (P < 0.01) in Simpsons diversity was observed with increasing levels of vegetable in the maize silage. Fungal diversity was not affected (P ≥ 0.13) by vegetable type, concentration, inoculant addition or interactions upon silage opening after 14 days of aerobic stability (Table 3).

**Figure 1:**
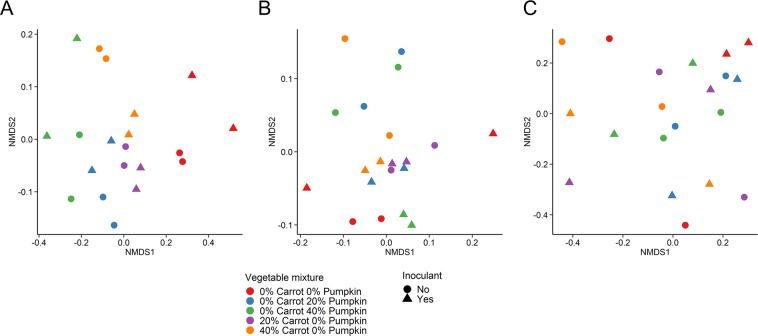
Non-metric dimensional scaling (NMDS) plot of the Bray-Curtis dissimilarities of the (**A**) bacterial microbiota in maize silage (Stress = 0.07), (**B**) fungal microbiota in maize silage (Stress = 0.13), and (**C**) fungal microbiota in silage following 14 d aerobic exposure (Stress = 0.18), by vegetable mixture and use of an inoculant.

**Figure 2:**
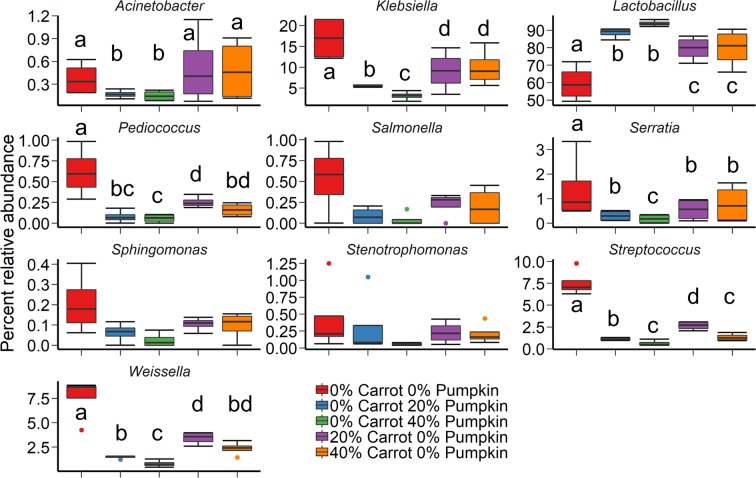
The 10 most relatively abundant bacterial genera in maize ensiled with carrots or pumpkin at 0, 20 or 40% DM, by vegetable mixture, after 70 d of ensiling. Different lowercase letters indicate significantly different means (P < 0.05).

The bacterial community structure of the silage was strongly affected by ensiling with either 20% or 40% carrot or pumpkin based on the Bray-Curtis dissimilarity between samples (Fig. 1A; R^2^ = 0.72; P ≤ 0.001). There was also a significant, but weak, effect from the use of an inoculant (R^2^ = 0.05; P = 0.03). All silages were dominated by *Lactobacillus* spp. after 70 days ensiling, regardless of vegetable (Fig. 2). However, there was a greater relative abundance of *Lactobacillus* spp. in the 40% carrot and 40% pumpkin silages, compared with the 100% maize silage (Supplementary Table 3). Members of the *Klebsiella, Pediococcus, Salmonella* and *Weissella* genera were more abundant in the 100% maize treatment.

**Figure 3:**
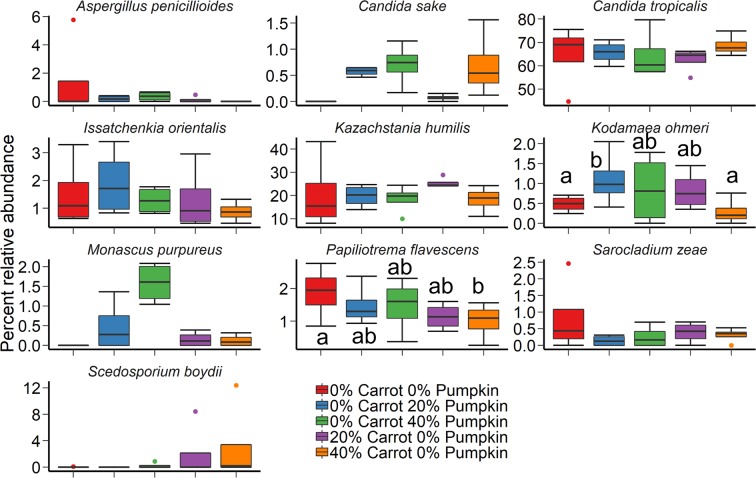
The 10 most relatively abundant fungal species in maize ensiled with carrots or pumpkin at 0, 20 or 40% DM, by vegetable mixture, after 70 d of ensiling. Different lowercase letters indicate significantly different means (P < 0.05).

The fungal community structure of maize silage was not affected by vegetable mixture (Fig. 1B; P ≥ 0.05), although there was an effect from the use of inoculant (R^2^ = 0.09; P = 0.03). Silage fungal microbiota was dominated by the yeast species *Candida tropicalis* across all treatments after 70 d ensiling (Fig. 3). The relative abundance of *Papiliotrema flavescens* was reduced in silage containing 40% pumpkin, compared with the control. In addition, the 20% carrot silage mixture had a higher relative abundance of *Kodamaea ohmeri* in comparison with the 100% maize silage (P < 0.05). No other differences were observed among the 10 most relatively abundant fungal species.

**Figure 4:**
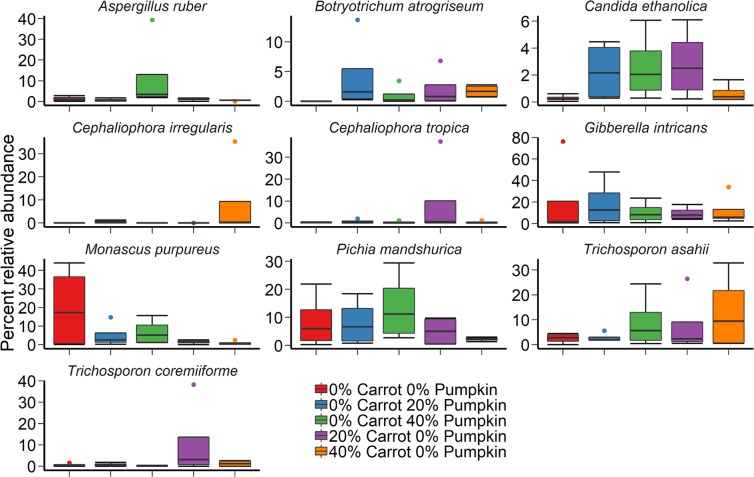
The 10 most relatively abundant fungal species in maize ensiled with either carrots or pumpkin at 0, 20 or 40% DM, by vegetable mixture, after day-14 of aerobic exposure.

The fungal community structure was not affected by either vegetable mixture (P ≥ 0.05) or the use of an inoculant (Fig. 1C; P ≥ 0.05) following the aerobic stability trial. In terms of the fungal microbiota of the maize silage, only *Monascus purpureus* was among the 10 most relatively abundant genera during silage and aerobic stability sampling (Fig. 4). The relative abundance of this species was highly variable, being more abundant in 40% pumpkin silage, compared to all other treatments, and on average present at 0–2% in vegetable treatments, compared to 40% in 100% maize silage samples following the aerobic stability trial.

Seven of the 10 most relatively abundant bacterial genera were influenced by vegetable treatment in crop maize combined with vegetables prior to ensiling (P < 0.05; Supplementary Fig. 1). However, there was no effect (P ≥ 0.05) of vegetable treatment on the relative abundance of *Acinetobacter, Gluconobacter* and *Lactobacillus* genera. The control maize treatment was mostly comprised of *Klebsiella*, *Streptococcus* and *Pediococcus* genera, similar to the 20% DM carrot treatment. Incorporation of carrot at 40% DM resulted in the largest relative abundance (P < 0.05) of *Weissella* genera, *Pseudomonas* genera the most relatively abundant in the 40% DM pumpkin treatment (P < 0.05).

### *In vitro* fermentation characteristics

No effects were observed (P ≥ 0.11) on pH or total VFA concentration of silage containing unsalable vegetables due to vegetable type, level or vegetable type × level. An increase (P = 0.01) in *in vitro* dry matter digestibility (IVDMD) was observed with increasing amounts of vegetable in the silage DM. A level × inoculant interaction was observed for maize silages when considering total gas production and CH_4_ (Table 4). As the level of pumpkin increased (P ≤ 0.01) in inoculated maize silage DM, CH_4_ concentration (expressed as mg/g DM or mg/g digestible DM) increased. Conversely, gas (mL/g DM) decreased (P = 0.02) as the level of pumpkin increased in maize vegetable silage treated with an inoculant.

**Table 4:** *In vitro* fermentation characteristics of maize silage ensiled with unsalable carrots or pumpkin after 24 h of incubation.

	0	Carrots		Pumpkin		SEM	P-values^1^						
		20	40	20	40		Veg	Level	Veg × Level	Inoculant	Level × Inoculant	L	Q
Gas mL/g DM	99.8	97.9	98.9	98.5	94.4	7.84	0.75	0.82	0.85	0.04	0.02	0.53	1.00
CH4, %	11.9	10.7	13.7	12.5	12.8	0.7	0.61	0.11	0.24	0.11	0.10	0.09	0.17
CH4, mg/g DM	9.0	7.7	9.9	9.1	9.3	0.87	0.61	0.2	0.29	0.02	0.002	0.43	0.12
CH4, mg/g DMD	16.4	13.1	16.8	16.8	17.1	1.47	0.2	0.27	0.28	0.01	0.001	0.65	0.13
pH	6.2	6.2	6.1	6.2	6.2	0.04	0.77	0.08	0.71	0.07	0.65	0.67	0.0281
IVDMD	55.8	59.6	59.5	54.5	54.4	1.7	0.01	0.68	0.19	0.12	0.29	0.47	0.62
Total VFA (mM)	96.3	93.1	95.3	95	96.4	3.26	0.39	0.28	0.78	0.86	0.17	0.77	0.13
**Volatile fatty acids (mmol/100 mmol)**													
Acetic (A)	58.6	59.2	58.7	58.6	59	0.53	0.67	0.42	0.24	0.23	<0.0001	0.3	0.42
Propionic (P)	27.2	27.2	27.5	27.5	26.9	0.41	0.71	0.9	0.44	0.42	0.03	0.92	0.66
Butyric	11.9	11	11.2	11.3	11.2	0.26	0.56	<0.01	0.72	0.91	0.47	0.001	0.05
Valeric	0.94	0.99	1.02	1.07	1.15	0.27	0.02	0.01	0.12	0.39	0.50	0.002	0.44
BCVFA	1.45b	1.51ab	1.57a	1.61c	1.73a	0.27	<0.01	<0.01	0.02	0.04	0.36	<0.0001	0.79
A:P	2.2	2.2	2.1	2.1	2.2	0.04	0.88	0.99	0.32	0.36	0.003	0.99	0.88

^1^P-values for Vegetable × inoculant >0.21; Means with different letters – a, b, c differ (P ≤ 0.05).

BCVFA, branched-chain volatile fatty acids (iso-butyric + iso-valeric).

Acetic acid and propionic acid percentages of total VFA (mmol/100 mmol) increased (P ≤ 0.03) in inoculated maize silage as vegetable inclusion increased (Table 4). Consequently, the ratio of acetic: propionic acids (A:P) in the inoculated vegetable maize silage shifted as acetic and propionic acids increased (P ≤ 0.01). The interaction between vegetable × level led to an increase (P = 0.02) in BCVFA percentage of total VFA, this increase also influenced by inoculant (P = 0.04) added to the silage DM.

## Discussion

The DM content of silage varies greatly, with forage type playing a major role in the rate and degree of fermentation^14^. As expected, the inclusion of unsalable vegetables to maize silage lowered DM content, and increased DM loss during the ensiling process, regardless of the vegetable type (Table 1). Loss of DM in silage occurs through microbial consumption of the forage during the fermentation phase, compounded here using vegetables at 20 or 40% DM due to the high moisture content, in agreement with previous studies^5,7,15^. However, despite an average DM of 29–35.3% at 20% inclusion and 22.4–31% DM at 40% vegetable inclusion, silage quality, as measured by the pH at opening (pH ≤ 4.0), was not affected. This aligns with Jalč *et al*.^16^ who demonstrated that high-moisture maize silages (25% DM) also yield a low pH of 3.55.

Vegetable addition to maize silage had no effect on silage surface temperature indicating longevity and quality of the silage was maintained. The use of a second-generation microbial inoculant improved the aerobic stability of silage after 14 days despite no effect of the inoculant on silage pH at opening. This indicates the high moisture content did not affect the fermentative capacity of the microbial community, in agreement with previous studies reporting microbial inoculants cause a rapid pH decline in the first 7 days of ensiling, followed by stabilization^5,17–19^. The inoculant used in this study contained a combination of *L. plantarum* and *L. buchneri*, well-known for rapidly decreasing silage pH to prevent microbial spoilage^20^. These findings indicate the benefits of applying the current inoculant to produce greater acetic acid concentrations, thus improving aerobic stability through the inhibition of yeast and mold growth upon aerobic exposure^21,22^.

Crude fat increased up to 3.9% DM in silage with 20 or 40% DM vegetable (40% DM with pumpkin), surpassing the mean crude fat content of 2.84% for maize silages from a meta-analysis of 234 Brazilian studies^23^. Higher dietary crude fat up to 6–8% provided to ruminants can increase overall energy intake, beyond that of VFA utilization or protein digestion^24^. However, no difference was observed for NDF contents (% DM) irrespective of vegetable or proportion of inclusion in the silage DM (Table 1). Despite the high moisture content of carrot and pumpkin, NDF content ranged from 9% (carrot) to 26% (pumpkin) for fresh vegetables on a DM-basis^7^. Maize ensiled with green beans have recorded a mean NDF content of 38–45% at 40% DM^25^, similar to values obtained within this study, ranging from 43.3–44.1% DM NDF for pumpkin and carrots respectively. Contrary to results from this study, NDF quantity has previously been associated with decreased VFA production, although related to organic matter digestibility rather than overall silage quality^26^.

The concentration of total VFA in a silage is not often used as an indicator of silage quality. However, silage that undergoes extensive fermentation is expected to yield a higher total VFA concentration^22^. In the present study, the addition of pumpkin yielded a 4 mM higher total VFA concentration than carrots, through a higher acetic acid concentration with pumpkin silage. Organic matter digestibility may influence VFA concentrations when comparing fresh vegetables or residue silages with a control crop silage^27^. The high organic matter digestibility of pumpkin pulp silages (60.65%) surpassed that of a maize silage (55.35%)^28^, which could explain the higher proportion of acetic acid, as part of total VFA concentration observed in vegetables silages in this study. Cao *et al*.^5^ and Yang *et al*.^17^ found waste vegetable residues including lettuce, Chinese, red and white cabbage contain epiphytic LAB similar to common livestock forages. Upon ensiling, vegetables with moderately high water-soluble carbohydrate (WSC) contents yielded high VFA concentrations, reduced silage pH and inhibited harmful aerobic bacteria, yeasts and molds^5,17^. Furthermore, Yang *et al*.^29^ noted the positive influence of the addition of a WSC in the form of glucose to wheat straw silage on acid production and the consequential lowering and stabilization of silage pH during fermentation.

Acetic acid concentrations of silages in this study represented over 90% of total VFA, but were not affected by silage inoculation. This was unexpected, as silages containing a 2^nd^ generation inoculant are known to yield a high concentration of acetic acid^18^. Typically, a distinct effect of *L. buchneri* is observed via increased conversion of lactic acid to acetic acid^18^ during the ensiling process. However, this effect may have been diluted by the action of pumpkin in the vegetable silage. Pumpkin, high in both sugars and pectin, provides nutrients that are rapidly consumed by bacteria upon fermentation^30^. A decrease in lactic acid, followed by an increase in acetic acid with 20 or 40% pumpkin on a DM-basis could explain the minimal effect of the inoculant in this study.

Incorporation of unsalable carrot or pumpkin at 20 or 40% DM into maize silage was directly correlated with increased bacterial diversity after 70 days of ensiling (Fig. 1). Diverse epiphytic microbial populations have been well documented on root vegetables in contact with the soil rhizosphere^31^. Previous reports have suggested that the epiphytic microbial populations present on carrots are highly diverse, when compared to other vegetables including tomatoes, onions and cucumber^32^. In this study, carrot inclusion influenced the epiphytic bacterial population of the vegetable silages being dominated by *Klebsiella*, *Weissella*, *Pseudomonas, Salmonella* and *Streptococcus* genera. Carrots can possess an endophytic bacterial population capable of promoting growth of the vegetable^33^ and increasing nitrogen assimilation into plant tissue^34^. However Surette *et al*.^35^ identified several genera on carrots, including *Agrobacterium* and *Variovirax*, bacteria typically isolated from soil^36,37^ that were not present in the current samples. This difference could be explained as a result of commercial washing of the vegetables with Sodium hypochlorite, which has been previously demonstrated to reduce bacterial counts of *Salmonella* and *Listeria* by 2 log at 200 mg/L^38^. However, the long-term efficacy of sodium hypochlorite to reduce surface pathogens on stored fresh produce is debated. Fresh carrots stored after washing with sodium hypochlorite had an increased coliform load of 3.1 log CFU/g following 6 days storage^39^. In agreement, the carrots ensiled in this study were stored for 3 days prior to ensiling resulting in the presence of *Klebsiella* being most prominent in silage with carrot inclusion at 20 and 40% compared to the control, suggesting increasing the vegetable content can increase microbial diversity. Similarly Peng, *et al*.^40^, observed an increase in bacterial richness with increasing vegetable content, as well as an increase in the number of fungal OTUs in the vegetable silage after 14 days aerobic exposure.

When comparing the inclusion of carrots versus pumpkin in maize silage after 14 days aerobic exposure, the greater bacterial richness observed with the inclusion of carrots could be explained by either the promotion of bacterial diversity by carrots, or the inhibition of bacterial proliferation with pumpkin inclusion. Previously^41^, pumpkin oil extract had no inhibitory effect on any microbes at a maximum concentration of 2.0% v/v, despite its rind exhibiting antifungal properties through the proteins Pr-1 and Pr-2^42,43^. Similarly, Park *et al*.^42^ determined that Pr-2 acted against pathogenic fungi via damage of the *Fusarium oxysporum* cell membrane, thereby inhibiting growth of the fungus. However, fungal diversity was not affected by vegetable type in the current study, suggesting that pumpkin inclusion did not elicit an antifungal effect. Overall, this suggest the likely disparity in diversity with carrot versus pumpkin inclusion in silage, was due to increased bacterial proliferation with carrot inclusion.

Two fungal species were prominent in the vegetable silage population at 70 d ensiling – *Candida tropicalis* and *Kazachstania humilis*. The fungus *C. tropicalis* is a heat-tolerant yeast that can produce ethanol under mildly acidic conditions^44^. During the ensiling process, heat production can elevate mean silage temperatures up to 12 °C above ambient temperature^45^. Hence, the presence of *C. tropicalis* in this study is not surprising, as it has previously been detected in whole maize silage alongside *C. quercitrusa*^46,47^. However, *C. quercitrusa* was not detected on the surface of maize silage after 70 days of ensiling in this study. Typically, the detection of both *C. tropicalis* and *C. quercitrusa* prior to ensiling and their resultant presence or absence can be attributed to their low tolerance of acetic acid^46^, the quantity of which increased at 40% pumpkin in the silage DM. Formerly *Candida humilis*^48^, *K. humilis* has been detected in silage at 45 or 90 d fermentation^49^. Other members of the *Kazachstania* genera are often detected on maize silage undergoing aerobic spoilage^50^. However, little is known about the action of *K. humilis* in the ensiling or aerobic exposure process, except that a higher relative abundance of *K. humilis* in this instance compared to other fungal species could be influenced by the external environment^51^.

When examining the silages *in vitro*, a decrease in CH_4_ production was expected as NDF content declined with 20 or 40% DM vegetables. However, an inhibitory effect on CH_4_ did not occur, but rather an increase in methane per gram digestible DM with the use of a silage inoculant was observed. The results corroborate those obtained by Rabelo *et al*.^52^ and Ellis *et al*.^53^, where an increase to 40% vegetables in inoculated maize silage increased gas production and thus, increased CH_4_ production up to 16.8 mg/g DM in the carrot treatment. The digestion of hemicellulose relative to cellulose in a feed can impact CH_4_ production^54^. More specifically, vegetables such as pumpkin have a hemicellulose:cellulose ratio equal to that of grasses, at 0.67:1 on a DM basis^55,56^ which could increase CH_4_ production upon *in vitro* incubation^56^. Nevertheless, this was the only observed effect on methane, in agreement with Hristov *et al*.^57^. *In vitro* fermentation of pumpkin residues has been shown to increase dry matter digestibility and decrease total gas production^58^, which could decrease total CH_4_ production proportionally. In this study, there were no effects of ensiled pumpkin on these parameters, which may have been influenced by the stage of maturity, and thus starch content of the crop maize^59^.

*In vitro* digestibility of carrots has been reported at 71.9%^60^, correlating with increased silage digestibility with carrot included at 20 or 40% DM in the current study. Pre-inoculated maize and wheat silages were reported to have increased NDF digestibility at 24 h *in vitro* incubation by up to 104 g/kg and 236 g/kg DM, respectively, compared to the control^61^. However, at 24 h *in vitro* incubation, treatment of ryegrass-clover silage using *L. plantarum*, *L. buchneri* and *Lactobacillus lactus* increased CH_4_ (mL/g of organic matter), attributed to increased gas production and digestibility^53^. This was supported by Rabelo *et al*.^62^, who identified that *L. buchneri* used as a silage inoculant increased *in vitro* gas production and rate of degradability in maize silage, through its preservation of highly digestible water-soluble carbohydrates (WSC). Consistent with the results from this study, Rabelo *et al*.^62^ ascertained that the proportion of acetic acid increased in maize silage treated with *L. buchneri*, similar to other experiments conducted into the action of *L. buchneri* on maize silages, where acetic acid and overall digestibility of inoculated silage increased in rams upon an *in vivo* study^63^. Laflamme^64^ noted that whole carrots in an alfalfa hay silage at a ratio of 3:1 carrot:hay produced silages of high quality – as observed through its pH of 4, low butyric acid ammonia-N concentrations, along with 63% DM digestibility. As expected, our study yielded lower digestibility and higher pH, given the carrot:hay ratio in the Laflamme^64^ study. As our study had a maximum inclusion level of 40% carrot or pumpkin, NDF concentration was also distinctly lower at 43.5% than the reported 56.2%^64^.

Overall, the results from this study suggest that carrot or pumpkin, combined with crop maize at 20 or 40% DM decreases bacterial richness and increases the inverse Simpson’s diversity index after 70 days ensiling. Such a modification following vegetable inclusion may be beneficial to silage quality, given the resulting dominance of the spoilage-inhibiting *Lactobacillus* spp. from opening to 14 d after aerobic exposure. Silage quality was improved with vegetable inclusion evidenced by greater *in vitro* digestibility. Although no mitigatory effects on *in vitro* CH_4_ production were observed in this study, concentrations of acetic acid, which represents over 90% of the total VFA, increased with carrot or pumpkin at 20 or 40% DM. Therefore, our results suggest unsalable carrots or pumpkin can be successfully ensiled with crop maize up to 40% DM to produce a high quality silage, with 40% DM inclusion of carrot producing the most desirable nutritional parameters and fermentation characteristics *in vitro*.

## Methods

### Silage production

Crop maize was harvested in January 2018 at The University of Queensland (UQ), Gatton, QLD (27°56′S, 152°28′E). Unsalable vegetables (carrot and pumpkin) were provided by Kalfresh, Kalbar QLD (27°94′S, 152°57′E) immediately after grading and transported using a refrigerated vehicle to Gatton within 24 hr, vegetables were ensiled within 3 days of grading. A second-generation silage inoculant (SI-LAC® EXTRA, Grevillia Ag, QLD, AUS), containing a combination of homolactic bacteria; *L. plantarum* and *E. faecium* and the heterolactic bacterium *L. buchneri* was used as the silage inoculant.

Maize was harvested over one day, resulting in 2 mini silos per treatment. Accordingly, 20 mini silos were constructed from PVC piping with an attached water valve, at dimensions of 90 mm diameter by 55 cm height and a volume of approximately 3500 cm^3^. Each mini silo was packed using a hydraulic press to a density of 240 kg/m^3^. Treatments comprised on a dry matter (DM) basis: (1) 100% crop maize, control; (2) 20% carrot with 80% crop maize; (3) 20% pumpkin with 80% crop maize; (4) 40% carrot with 60% crop maize; or (5) 40% pumpkin with 60% crop maize; where each treatment was replicated to facilitate a control, no inoculant, compared to an inoculant treatment. Each silo was weighed immediately after closing.

### Opening of mini silos

#### Silage sampling

After 70 days of ensiling, each mini silo was weighed and recorded prior to opening. The top 3–4 cm of silage was disposed from each mini silo due to microbial spoilage. The contents of each mini silo were mixed well, and 100 g samples were immediately frozen in liquid nitrogen and stored at −20 °C for subsequent analyses.

#### Dry matter content and dry matter loss

Approximately 250 g of silage from each individual mini silo were used for determination of DM content. Trays were dried at 55 °C for 48 h. DM loss was calculated as the difference between mini silo wet weight at time of ensiling, and mini silo weight at opening.

#### pH

A sub-sample of silage (15 g) was blended with 135 g of distilled water to produce a dilution of 1:10, for 30 seconds at room temperature and then filtered through double-layered cheesecloth. Approximately 10–15 mL of filtrate was used to determine pH (Activon Model 209, Gladesville, NSW, Australia).

### Organic acids, VFA and ethanol

Forty millilitres of filtrate were placed on ice for VFA analyses as described by Zahiroddini *et al*.^65^. Briefly, samples were centrifuged at 10,000 × *g* for 15 min at 4 °C. Supernatant (5 mL) was combined with 1 mL 25% (wt/vol) metaphosphoric acid in a ratio of 5:1, and frozen at −20 °C, until analysis by gas-liquid chromatography. Ethanol was determined as previously described by Kudo *et al*.^66^ on an Agilent technologies 7820A gas-liquid chromatograph system, using a DB-FFAP column of dimensions 30 m × 0.32 mm × 1.00 µm.

### Aerobic stability

Approximately 500 g of silage per mini silo was placed into two aluminium trays and exposed to ambient temperature for 14 days. Thermal images of each tray were taken from 1 m for 14 consecutive days at 0900 and 1600, using a FLIR E50 Thermal Imaging Camera (FLIR, Wilsonville, OR, United States of America) and FLIR Tools software. The images provided estimates of the mean, minimum and maximum temperatures for each tray per day, whilst visual assessment of microbial spoilage of the silage was also made each day and recorded. After the 14-day period, the silage samples for each treatment were thoroughly mixed and a sample of 70 g was collected for DNA extraction of aerobically exposed silage. Samples were frozen in liquid nitrogen and stored at −20 °C until DNA extraction.

### DNA extraction

Preparation of samples for DNA extraction was conducted as previously described by Yu and Morrison^67^. Briefly, 300 mg of silage was homogenised with 1.1 mL of InhibitEx buffer. Following bead beating, incubation and centrifugation, 600 µL of supernatant was added to 25 µL Proteinase-K. Samples were loaded into a QIAGEN QIAcube (Qiagen, Hilden, Germany) for DNA extraction, programmed with the QIAamp Fast DNA Mini Stool Kit protocol. The loaded samples were automatically digested, bound and centrifuged with a spin column, washed twice and eluted. Determination of DNA yield was conducted using a NanoDrop 2000c spectrophotometer (Thermo Fisher Scientific, Wilmington, DE, United States of America) as described by Desjardins and Conklin^68^. Purity and integrity values were obtained through methods identified by Henderson, *et al*.^69^.

### Sequencing and analysis of the bacterial 16S rRNA gene and fungal ITS1 region

The V4 region of the archaeal and bacterial 16S rRNA gene was amplified as previously described^70^ using the 515 f Modified (5′-GTGYCAGCMGCCGCGGTAA-3′) and 806r modified (5′-GGACTACNVGGGTWTCTAAT-3′) primer sequences^71^. The primers ITS1F (5′-CTTGGTCATTTAGAGGAAGTAA-3′) and ITS2 (5′-GCTGCGTTCTTCATCGATGC-3′)^72^ were used to amplify the ITS1 region of fungi. Both 16S rRNA gene and ITS1 sequences were sequenced using the MiSeq Reagent Kit v2 (500 cycles; Illumina, Inc., San Diego, CA, USA) and an Illumina MiSeq instrument.

DADA2 v. 1.8^73^ was used in R v. 3.5.1 to process the 16S rRNA gene and ITS1 sequences. Briefly, forward and reverse 16S rRNA gene sequences were trimmed to 220 and 200 bp, merged, and then chimeras were removed. Taxonomy was assigned to the remaining sequences, referred to here as OTUs at 100% similarity, using the RDP naïve Bayesian classifier and the SILVA SSU database release 132^74^. For ITS1 sequences, reads were quality-filtered using the default parameters and a minimum length of 50 bp but were not trimmed to the same length. The reads were merged, chimeras removed, and taxonomy assigned to the ITS1 sequences using the RDP naïve Bayesian classifier and the UNITE database v. 8.0^75^. The number of OTUs per sample, Shannon diversity index, and inverse Simpson’s diversity index for 16S rRNA gene and ITS1 datasets were calculated in R using Phyloseq v. 1.26.0^76^. Bray-Curtis dissimilarities were calculated using vegan 2.5–3^8,77^ in R. Prior to analysis, the silage 16S, silage ITS1, crop 16S, and the aerobic stability ITS1 OTU tables were randomly subsampled to 4,450, 2,000, 1,000, and 6,000 sequences per sample, respectively, prior to analysis of alpha- and beta- diversity. All 16 S rRNA gene and ITS1 sequences were submitted to the sequence read archive under BioProject accession PRJNA525850.

### *In vitro* and rumen fermentation characteristics

The *in vitro* experiment was carried out at the University of Queensland Gatton campus. The steers used in this study were cared for under the approval and guidance of the University of Queensland Animal Ethics Committee (AE35581), in accordance with the Animal Care and Protection Act (2001). Two rumen-cannulated Droughtmaster steers had access to *ad libitum* pasture supplemented daily with 3 kg carrot and pumpkin each (up to a maximum of 30% DM) for one week prior to the *in vitro* study. Rumen fluid was obtained from both steers 2 h post-feeding, rumen fluid collection occurring in the dorsal, anterior ventral, medium ventral, posterior dorsal and posterior ventral regions of the rumen. The pooled rumen fluid was strained through 4 layers of cheesecloth into a pre-warmed, insulated 1 L Thermos and returned to the laboratory promptly. Methodology details for *in vitro* incubations and chemical analysis were provided in Williams *et al*.^78^.

### Statistical analyses

Statistical analyses of the nutrient composition, organic acid and aerobic stability data were obtained using a PROC MIXED SAS^79^, with tabulated results provided as least squares mean and standard error of mean (SEM). The data was analysed as a completely randomized design, with the fixed effects being; vegetable type (carrot vs. pumpkin), level (i.e.: the level of vegetables on a DM basis in the sample; 0, 20 and 40%) and the interaction between vegetable type and level, whilst the random effect was the mini silo. As the maize silage was harvested on the single day, with 2 replicates per treatment resulting in a total of 20 mini silos, the experimental unit for maize treatments was represented by each mini silo.

Normality of the data was determined using a PROC UNIVARIATE SAS. Orthogonal polynomial contrasts were also used to evaluate the linear and quadratic responses to the differing concentrations of carrots or pumpkins (0, 20, or 40% on a DM basis) in the various silage treatments (e.g. maize, inoculant or non-inoculant). Differences among means were determined using a least square linear hypothesis test, with the significance level for all data declared at P ≤ 0.05.

### Appropriateness of research

This work is within the scope of Scientific Reports, as it provides novel evidence toward the efficacy of forage replacement with unsalable vegetables to create a livestock feed, which would otherwise become waste through conventional disposal methods. The study not only has implications on current knowledge of microbial ecology, nutritive characteristics, aerobic stability in silage, but also societal implications, particularly as food security becomes an increasingly pertinent issue worldwide. Provision of the findings from this study will also allow readers to consider alternative methods of feeding livestock, with the view of further encouraging sustainability in animal production.

## Supplementary information

Supplementary Information

## Data Availability

All 16S rRNA gene and ITS sequences were submitted to the Sequence Read Archive under BioProject number PRJNA525436.
