# Supplementary material for: Microbial characterization and fermentative characteristics of crop maize ensiled with unsalable vegetables
Source: Sci Rep. 2019 Sep 12;9:13183. doi: 10.1038/s41598-019-49608-w (PMC6742658; doi:10.1038/s41598-019-49608-w)
Supplement: Supplementary file 1 — Supplementary Information [file 41598_2019_49608_MOESM1_ESM.pdf]

Microbial characterization and fermentative characteristics of crop maize ensiled with unsalable vegetables

Kristian Hooker[1], Daniel L. Forwood[1,4], Eleonora Caro[1,2], Yuxin Huo[1], Devin B. Holman[3], Alex V. Chaves[1], Sarah J. Meale[4*]

[1]School of Life and Environmental Sciences, Faculty of Science, University of Sydney, Camperdown, NSW, Australia

[2]Department of Agricultural, Forestry and Food Science, University of Turin, Torino, TO, Italy

[3]Lacombe Research and Development Centre, Agriculture and Agri-Food Canada, Lacombe, AB, Canada

[4]School of Agriculture and Food Sciences, Faculty of Science, University of Queensland, Gatton, QLD, Australia

*s.meale@uq.edu.au

Supplementary Table 1. Chemical composition of maize crop at harvest and chemical compositions of fresh carrot and pumpkin

Parameter	**Maize crop**	**Carrot**	**Pumpkin**
Dry matter content (%)	36.9	9.7	12.6
Crude protein (% of DM)	7.9	4.8	10.7
aNDFom (% of DM)	63.2	11.1	18.1
NDF (% in DM)	45.99	14.3	8.8
Ash (% of DM)		5.1	6.2
Fat (% of DM)	2.16	16.1	19.2

Supplementary Table 2. Temperature difference between the environment and on the maize silage surface once exposed to oxygen from day 1 to day 14.

		Carrots			Pumpkin			P-values[1]				
	**Day**	**0%**	**20%**	**40%**	**20%**	**40%**	**SEM**	**Veg**	**Level**	**Veg × Level**	**Linear**	**Quadratic**
**Difference (ºC)**	**1**	-2.54	-2.54	-2.70	-2.66	-2.74	0.11	0.56	0.27	0.87	0.12	0.76
	**2**	-1.24	-1.47	-1.46	-1.72	-1.71	0.32	0.51	0.46	0.90	0.29	0.51
	**3**	-2.13	-2.07	-2.65	-2.25	-2.19	0.16	0.51	0.18	0.18	0.09	0.43
	**4**	-1.19	-1.17	-1.56	-1.50	-1.34	0.14	0.77	0.20	0.17	0.08	0.89
	**5**	-1.18	-1.16	-1.59	-1.23	-1.26	0.22	0.63	0.46	0.64	0.26	0.58
	**6**	-0.78	-0.79	-0.77	-0.91	-0.90	0.19	0.60	0.92	0.93	0.77	0.79
	**7**	-1.40	-1.35	-1.45	-1.29	-1.18	0.21	0.53	0.91	0.79	0.71	0.84
	**8**	-0.29	-0.26	-0.41	-0.58	-0.50	0.10	0.13	0.26	0.32	0.12	0.58
	**9**	-0.01	-0.05	-0.11	-0.31	-0.14	0.19	0.53	0.66	0.77	0.55	0.50
	**10**	-1.05	-1.20	-1.26	-1.23	-1.09	0.17	0.75	0.59	0.81	0.45	0.48
	**11**	-0.97	-0.97	-1.06	-1.16	-1.08	0.20	0.66	0.84	0.87	0.61	0.77
	**12**	-0.66	-0.66	-0.78	-0.74	-0.88	0.15	0.62	0.46	0.94	0.23	0.71
	**13**	-0.09	0.01	-0.11	-0.14	0.02	0.12	0.95	0.92	0.52	0.69	0.99
	**14**	0.47	0.31	0.42	0.18	0.43	0.14	0.72	0.27	0.86	0.73	0.12

[1]P-values for the treatments: Vegetable, carrot or pumpkin; Level, proportion of vegetable in silage on DM basis; Vegetable × Level, interaction. Statistical significance declared at P≤0.05.

Supplementary Table 3. Differentially abundant OTUs in the bacterial microbiota of either the 100% maize silage and 40% carrot or 100% maize and 40% pumpkin silage. BaseMean represents the normalized OTU abundance among all samples compared. Negative log2 fold change values indicate lower abundance in the 100% maize silage. Only differentially abundant OTUs with an adjusted p-value of less than 0.01 are included.

Treatment	BaseMean	log_2 fold change	Adjusted p-value	Genus
**100% maize vs. 60% maize 40% carrot**				
	156.66	-11.27	2.11×10^{-8}	*Lactobacillus*
	54.20	-9.74	6.97×10^{-4}	*Lactobacillus*
	40.57	-9.32	1.83×10^{-2}	*Lactobacillus*
	23.89	-8.56	4.00×10^{-2}	*Lactobacillus*
	156.44	-8.44	6.63×10^{-3}	*Lactobacillus*
	264.11	-5.71	6.63×10^{-3}	*Lactobacillus*
	9271.38	-5.47	6.78×10^{-7}	*Lactobacillus*
	348.41	-4.00	4.52×10^{-2}	*Lactobacillus*
**100% maize vs. 60% maize 40% pumpkin**				
	8190.23	-14.99	9.64×10^{-23}	*Lactobacillus*
	145.95	-11.88	1.98×10^{-14}	*Lactobacillus*
	35.82	-9.85	5.08×10^{-3}	*Lactobacillus*
	31.49	-9.67	5.19×10^{-3}	*Lactobacillus*
	122.50	-7.45	6.77×10^{-5}	*Lactobacillus*
	6632.69	-5.65	1.57×10^{-10}	*Lactobacillus*
	404.90	-4.89	2.74×10^{-3}	*Lactobacillus*
	384.69	-2.34	6.59×10^{-3}	*Lactobacillus*
	26.37	7.15	4.95×10^{-5}	*Weissella*

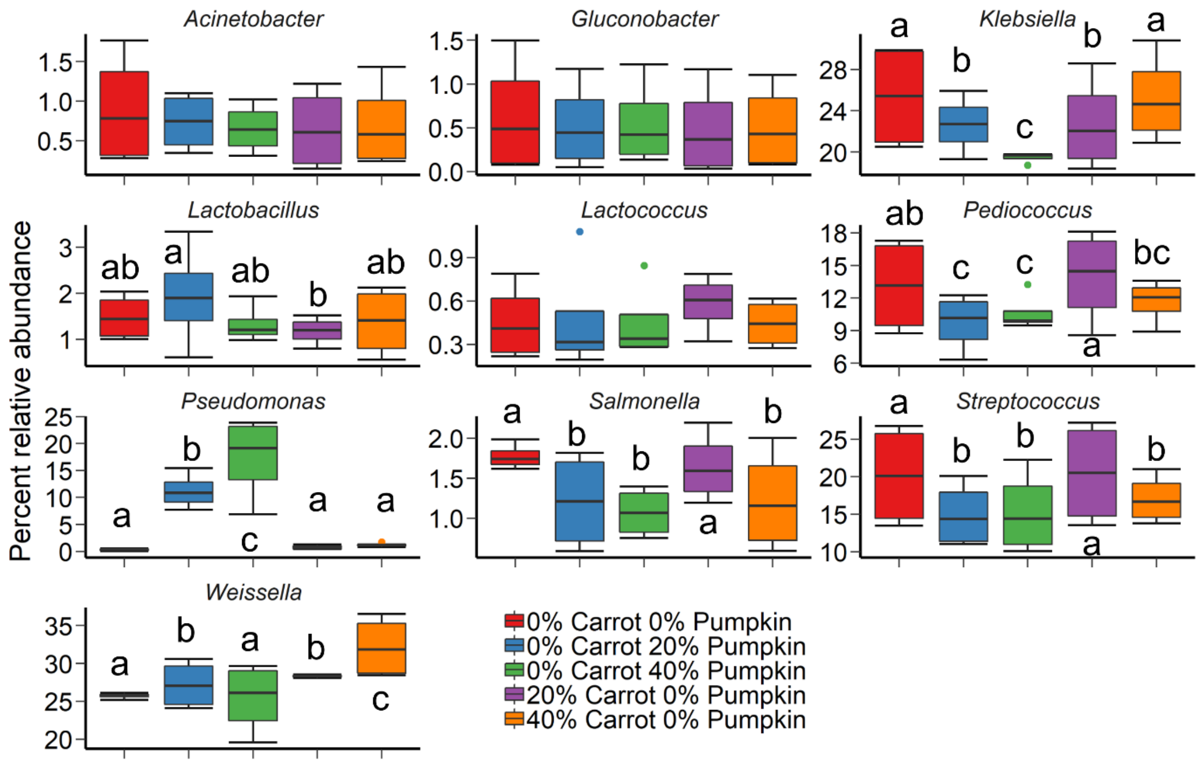

Supplementary Figure S1. The 10 most relatively abundant bacterial genera in maize prior to ensiling with either carrots or pumpkin at 0, 20 or 40% DM, by vegetable mixture. Different lowercase letters indicate significantly different means (P<0.05).
